# Resident-Led Clinical Documentation Improvement in Cardiology: Early Effects on Risk-Adjusted Quality Metrics

**DOI:** 10.1016/j.jaccas.2026.109869

**Published:** 2026-09-16

**Authors:** Akshay Raut, Shubhendu Bajpai, John Pamula

**Affiliations:** Department of Internal Medicine, Guthrie/Robert Packer Hospital, Sayre, Pennsylvania, USA

**Keywords:** acute heart failure, cardiovascular disease, health care economics

## Abstract

**Background:**

Risk-adjusted quality metrics, including the mortality index and length of stay index, are central to evaluating cardiovascular care, yet documentation gaps can misrepresent institutional performance.

**Project Rationale:**

At our rural academic medical center, the Cardiology service line demonstrated a baseline mortality index of 1.40, indicating 40% higher-than-expected mortality. We identified documentation gaps in common cardiology diagnoses as contributing factors.

**Project Summary:**

This quality improvement study, with a retrospective preintervention comparison, analyzed 2,143 Cardiology encounters over 18 months (July 2024-December 2025). A resident-led intervention including resident training, peer review, and clinical documentation improvement specialist collaboration was associated with mortality index improvement from 1.40 to 0.97 (30.7% relative improvement) and length of stay index improvement from 0.91 to 0.85 (6.6%).

**Take-Home Messages:**

A resident-led clinical documentation improvement initiative improved documentation accuracy of patient risk, increasing expected values and correcting artificially elevated quality indices without changing clinical care or actual patient outcomes.

## Visual Summary

**Figure:**
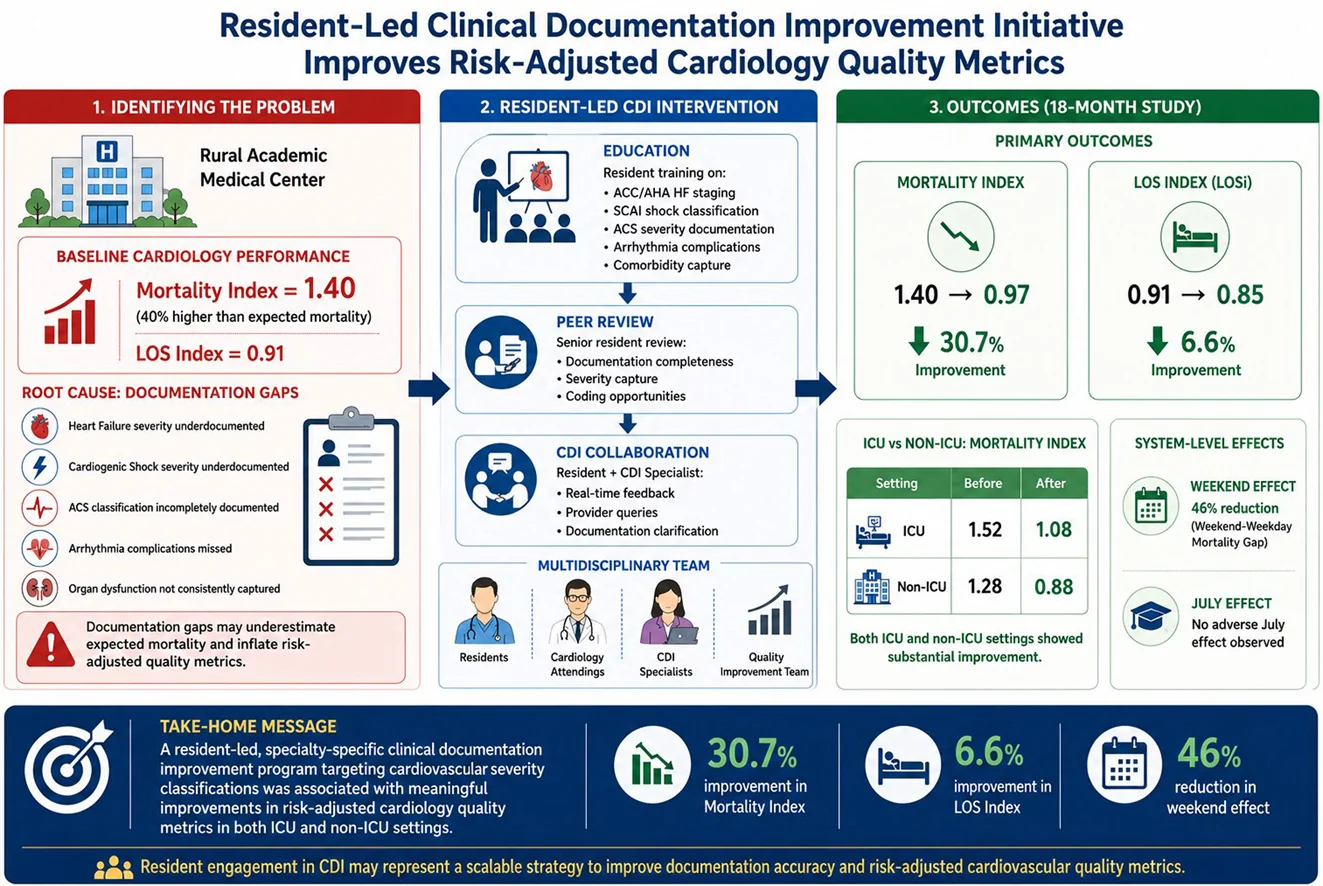

The mortality index and length of stay index (LOSi), which compare observed with expected mortality rates and length of stay, respectively, are key quality indicators as these indices affect Medicare Severity Diagnosis-Related Groups reimbursement, Hospital Value-Based Purchasing incentive payments, and public quality rankings.^1^^,^^2^ These metrics rely on implantable cardioverter-defibrillator-coded data from clinical documentation.^3^^,^^4^ Medical coders can only submit a diagnosis for reimbursement if the clinical team clearly documents it, even if the same diagnosis could be easily inferred from lab results or hemodynamic data. For this purpose, hospitals often have a dedicated Clinical Documentation Improvement (CDI) Team that assists the clinical team to improve clinical documentation. CDI initiatives do not alter clinical care delivery or actual patient outcomes. Rather, they improve the accuracy with which existing patient complexity is captured in the medical record, thereby increasing the calculated expected values and correcting the artificially elevated indices. Previous studies showed that CDI initiatives improve quality metrics.^1^^,^^2^^,^^4^ However, initiatives led by residents that specifically target cardiology severity classifications have not been thoroughly described.

## Case Summary Prompting the Project Launch

We found that the Cardiology service line Mortality Index at Robert Packer Hospital, a rural academic medical center in the Guthrie Health System, consistently exceeded 1.0, with a baseline of 1.40 indicating 40% higher than expected mortality, despite no identified deficiencies in care delivery. Meanwhile, the LOSi remained below 1.0 at 0.91. This paradox suggested that documentation gaps were artificially reducing expected mortality calculations rather than reflecting actual care quality deficiencies.

## Project Rationale

The cardiology service line was prioritized for CDI intervention for 3 reasons. First, established severity classification systems, such as American College of Cardiology (ACC)/American Heart Association heart failure stages, Society for Cardiovascular Angiography and Interventions cardiogenic shock classification, and ST-segment elevation myocardial infarction (STEMI)/non-ST-segment elevation myocardial infarction/unstable angina classification, directly relate to risk-adjustment variables that residents could address.^5^ Second, the high volume of cardiology admissions, both in intensive care unit (ICU) and non-ICU settings, meant that improvements in documentation would have a significant cumulative impact. Third, the project fit with institutional goals to enhance Vizient Clinical Database benchmarks and was motivated by the direct link between accurate documentation and value-based reimbursement.

Prior published evidence supports this approach.^1^^,^^2^ Seligson et al demonstrated a 25.4% decrease in the Mortality Index and a 5.6% increase in case mix index (CMI) on a heart and vascular service line through standardized documentation templates.^1^ These studies showed that multidisciplinary CDI teams, consisting of physicians, residents, and CDI specialists, were effective, a model similar to our approach.

## Project Description

This prospective IRB-approved quality improvement study with a retrospective pre-intervention comparison was carried out at Guthrie Robert Packer Hospital over 18 months, from July 2024 to December 2025. All adult patients admitted to the Cardiology service line, including both ICU and non-ICU settings, were included.

The project was conducted in 2 phases. Phase A, conducted from July 2024 to June 2025, involved collecting baseline data, with expected values sourced from the Vizient Clinical Database.

Pre-intervention chart review identified specific documentation gaps across several categories. For heart failure, “heart failure exacerbation” was frequently documented instead of “acute on chronic systolic heart failure with reduced ejection fraction, NYHA functional class III,” and American College of Cardiology/American Heart Association stage classification and Society for Cardiovascular Angiography and Interventions cardiogenic shock grading were often absent despite clinical evidence. For comorbid diseases, acute kidney injury meeting kidney disease improving global outcomes criteria was present in laboratory data but not explicitly diagnosed or staged; chronic kidney disease lacked stage specification; protein-calorie malnutrition was underdocumented despite qualifying body mass index or albumin values; and anemia of chronic disease and diabetic end-organ complications (nephropathy, neuropathy) were documented without specificity. For acute coronary syndromes, type 2 myocardial infarction was not distinguished from myocardial injury, and STEMI/non-ST-segment elevation myocardial infarction classification was absent in discharge documentation.

In Phase B, from July 2025 to December 2025, the resident-led CDI intervention was implemented. This included: 1) formal training for residents on cardiology-related and comorbid risk variables; 2) senior resident reviews of clinical documentation; and 3) collaboration with CDI specialists for clarification of documentation through provider queries.

The multidisciplinary team included internal medicine residents, CDI specialists, and quality improvement faculty. The main outcomes were LOSi and Mortality Index. Secondary outcomes included CMI. The analysis involved matched 6-month pre-post comparisons, interrupted time series (ITS) analysis, and stratified analyses based on ICU status, weekday/weekend admissions, July effect, and holiday periods (Thanksgiving, Christmas, and New Year's Day).^6^^,^^7^

## Project Deliverables

We analyzed a total of 2,143 Cardiology encounters, with 1,069 before the intervention and 1,074 after.

The Mortality Index shifted from being 40% higher than expected to 3% lower than expected. The complication or comorbidity/major complication or comorbidity capture rate increased from 41% to 49%, showing improved documentation accuracy. CMI remained stable, moving from 1.89 to 1.83, indicating that the improvements reflected better documentation, not population changes, although we cannot exclude temporal confounding (Table 1).^8^

**Table 1:** Primary and Secondary Outcomes: Pre-intervention vs Post-intervention

Metric	Pre-intervention	Post-intervention	Change
Mortality Index	1.40	0.97	−0.43 (30.7% improvement)
LOSi	0.91	0.85	−0.06 (6.6% improvement)
CC/MCC capture rate	41%	49%	+8 percentage points
Case mix index	1.89	1.83	−0.06 (stable)

CC/MCC = complication or comorbidity/major complication or comorbidity; LOSi = length of stay index.

Non-ICU patients showed a larger relative improvement in the Mortality Index (31.3%) compared with ICU patients (28.9%). The decreasing gap between ICU and non-ICU documentation (0.24-0.20) suggests improved documentation equity across different care settings (Table 2).

**Table 2:** ICU Stratification and Documentation Accuracy

Metric	ICU Pre	ICU Post	Non-ICU Pre	Non-ICU Post
Mortality index	1.52	1.08 (−28.9%)	1.28	0.88 (−31.3%)
LOSi	0.93	0.95	0.79	0.76 (−3.8%)
ICU–non-ICU mortality gap	0.24	0.20 (−17%)	—	—

ICU = intensive care unit; other abbreviations as in Table 1.

## Project Outcome, Impact, and Future Directions/Next Steps

The ITS analysis showed a 17% immediate improvement in LOSi at the start of the intervention, with a monthly decline in mortality of 0.15 post-intervention. This represented faster improvement compared with the preintervention period. Although ITS analysis strengthens causal links more than simple before-and-after comparisons, it doesn't fully eliminate the influence of concurrent trends or cointerventions.^6^, ^7^, ^8^

Pre-intervention weekend admissions had higher mortality indices (1.58 vs 1.32), reflecting the “weekend effect” noted by Itani et al^9^ (inpatient mortality OR: 1.07). After the intervention, the gap in mortality between weekends and weekdays narrowed by 46%. We found no negative July effect; and also found improvement during holiday periods (Table 3).

**Table 3:** Weekend Effect, July Effect and Holiday Effect Mitigation

Stratum	Pre-Mortality Index	Post-Mortality Index	Improvement	Gap Change
Weekday	1.32	0.92	30.3%	—
Weekend	1.58	1.06	32.9%	Gap: 0.26 → 0.14 (−46%)
Month of July	1.72	0.75	56.4%	—
Other Months	1.39	0.98	29.5%	
Holiday Period	1.44	1.00	30.6%	
Non-Holiday Period	1.38	0.95	31.1%	—

**Visual Summary:**
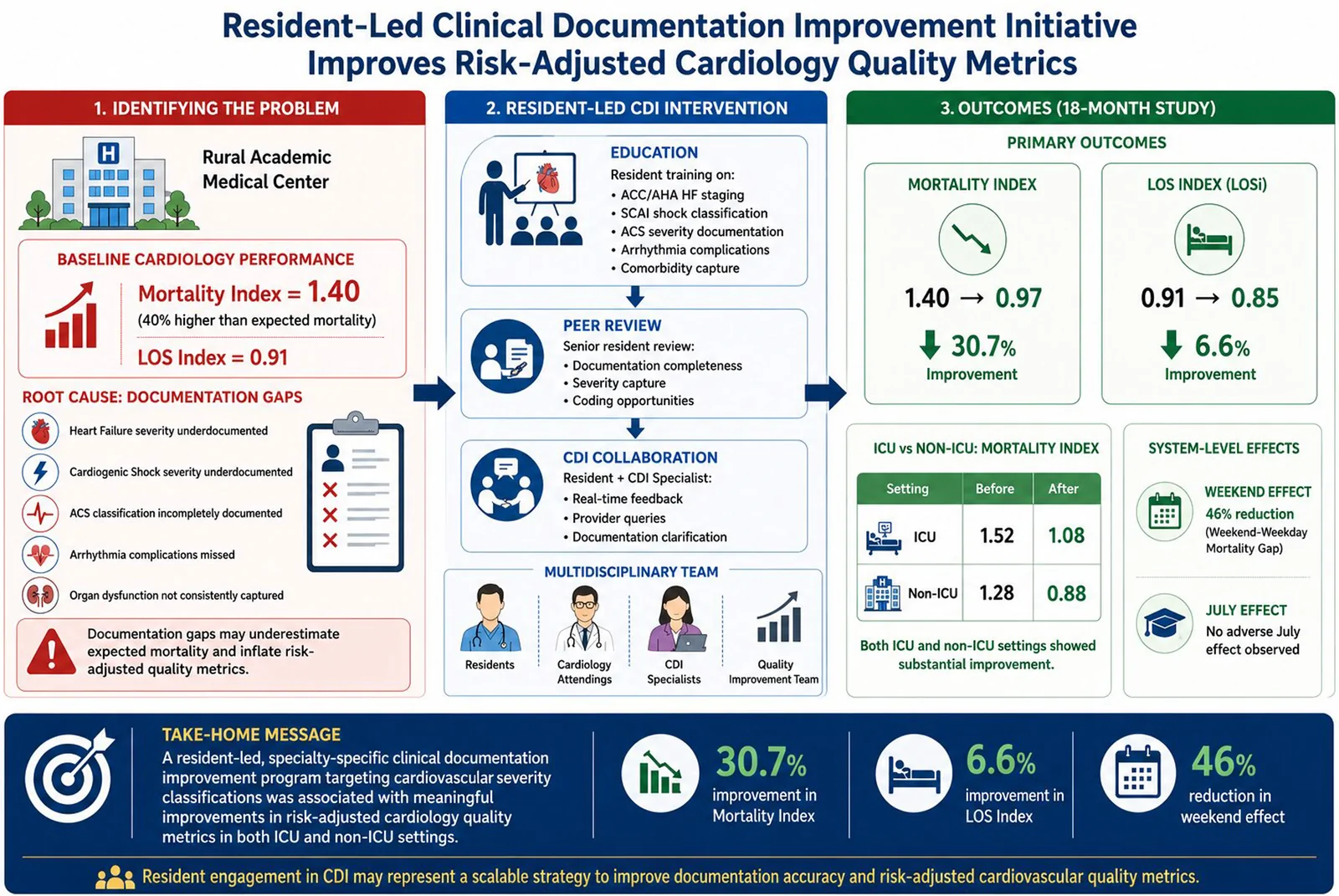
Resident-Led Clinical Documentation Improvement (CDI) Initiative Across the Cardiology Service Line Following the identification of documentation gaps, residents received specialty-focused education, participated in peer review, and collaborated with CDI specialists to improve documentation accuracy. The intervention was associated with improved capture of patient severity, resulting in a 30.7% improvement in mortality index and a 6.6% improvement in length of stay index, without changes in clinical care or patient outcomes. ACC/AHA = American College of Cardiology/American Heart Association; ACS = acute coronary syndrome; HF = heart failure; ICU = intensive care unit; LOS = length of stay; LOSi = length of stay index; SCAI = Society for Cardiovascular Angiography and Interventions.

Accurate documentation supports proper risk assessment and aids clinical decision-making for patients. For providers, the resident-led model fosters documentation skills early in their training. For payers, accurate Medicare Severity Diagnosis-Related Groups assignment ensures fair reimbursement, and for society, accurate metrics allow true comparisons between hospitals. Several steps could strengthen the evidence. First, validation across multiple sites through the Cardiovascular Quality Improvement and Care Innovation Consortium would assess applicability in different health systems.^7^ Second, linking to the American College of Cardiology National Cardiovascular Data Registry would allow us to connect documentation improvements to patient outcomes, such as readmission rates and postdischarge mortality. Third, we need to assess the sustainability of improvements beyond 6 months. Finally, developing automated EHR documentation prompts might help maintain improvements without relying solely on individual training efforts.

## Discussion

This resident-led CDI project resulted in a 30.7% improvement in the Mortality Index for the Cardiology service line. The increase in the complication or comorbidity/major complication or comorbidity capture rate from 41% to 49% indicates that the improvements in metrics were partly due to better documentation accuracy, rather than changes in the patient population or other trends. It bears emphasis that this initiative did not change clinical care, treatment protocols, or actual patient mortality. The observed improvement in the Mortality Index resulted entirely from more accurate documentation of pre-existing patient complexity, which increased the expected mortality denominator in the index calculation.

However, several factors should be considered when interpreting these results. The uncontrolled design could not rule out temporal confounding, Hawthorne effects, or concurrent changes in the institution. Although ITS analysis provides stronger causal inference by accounting for existing trends, it does not rule out the influence of cointerventions during the study.^7^ The steady CMI suggests, but does not prove, that documentation improvement was the main reason for the observed changes. Notably, non-ICU patients had greater relative improvement (31.3%) than ICU patients (28.9%). The 46% reduction in the weekend-weekday mortality gap is also significant. Our findings suggest that some of the “weekend effect” in cardiology quality metrics may stem from documentation issues, though differences in care delivery on weekends likely also play a role.^9^

The LOSi improvement from 0.91 to 0.85 warrants cautious interpretation. Although a lower LOSi may partly reflect documentation-driven recalibration of expected values, it also indicates patients were discharged earlier than expected. In heart failure, Sud et al^10^ demonstrated a U-shaped association between hospital length of stay and cardiovascular readmission, with both short and prolonged stays associated with higher readmission rates.

Limitations include a single-center design, which might limit generalizability, reliance on aggregate data rather than individual chart audits, potential temporal confounding in uncontrolled pre-post designs, a relatively short intervention period of 6 months, and the lack of direct patient-centered outcome measurements.

## Conclusions

This resident-led CDI initiative was associated with a 30.7% mortality index improvement and 6.6% LOSi improvement on the Cardiology service line. The intervention was associated with reduced day-to-day variation, mitigated weekend effects (46% gap reduction), and no adverse July Effect. While the uncontrolled design limits causal attribution, the improved risk-adjusted metrics support documentation accuracy as a primary mechanism. Multisite validation and linkage to patient-centered outcomes are needed to confirm generalizability.

## Funding Support and Author Disclosures

The authors have reported that they have no relationships relevant to the contents of this paper to disclose.

**Take-Home Messages**

• A resident-led clinical documentation improvement initiative targeting established cardiovascular severity classifications: heart failure staging, Society for Cardiovascular Angiography and Interventions cardiogenic shock criteria, and acute coronary syndrome classification was associated with a 30.7% Mortality Index improvement and 6.6% length of stay index improvement.
• This multifaceted approach combining specialty-specific education, peer review, and clinical documentation improvement collaboration offers a reproducible model for academic medical centers, though multisite validation and linkage to patient-centered outcomes are needed to confirm generalizability.
